# Phytochemical Profile and In Vitro Cytotoxic, Genotoxic, and Antigenotoxic Evaluation of *Cistus monspeliensis* L. Leaf Extract

**DOI:** 10.3390/ijms252413707

**Published:** 2024-12-22

**Authors:** Ghanya Al-Naqeb, Gianluca Zorzi, Amanda Oldani, Alberto Azzalin, Linda Avesani, Flavia Guzzo, Alessia Pascale, Rachele De Giuseppe, Hellas Cena

**Affiliations:** 1 Laboratory of Dietetics and Clinical Nutrition, Department of Public Health, Experimental and Forensic Medicine, University of Pavia, 27100 Pavia, Italy; rachele.degiuseppe@unipv.it (R.D.G.); hellas.cena@unipv.it (H.C.); 2 NBFC, National Biodiversity Future Center, 90133 Palermo, Italy; gianluca.zorzi@univr.it (G.Z.); linda.avesani@univr.it (L.A.); flavia.guzzo@univr.it (F.G.); 3 Department of Food Sciences and Nutrition, Faculty of Agriculture Food and Environment, University of Sana’a, Sana’a P.O. Box 1247, Yemen; 4 Department of Biotechnology, University of Verona, 37134 Verona, Italy; 5 PASS-Bio Med, Centro Grandi Strumenti, University of Pavia, 27100 Pavia, Italy; amanda.oldani@unipv.it; 6 Department of Biology and Biotechnology, University of Pavia, 27100 Pavia, Italy; alberto.azzalin@unipv.it; 7 Department of Drug Sciences, Section of Pharmacology, University of Pavia, 27100 Pavia, Italy; alessia.pascale@unipv.it; 8 Clinical Nutrition Unit, ICS Maugeri IRCCS, 27100 Pavia, Italy

**Keywords:** *Cistus monspeliensis*, phytochemicals, genotoxicity, antigenotoxicity, ImageStreamX imaging flow cytometer

## Abstract

*Cistus monspeliensis* L. (*C. monspeliensis*) is used in Italian folk medicine. This study was performed to determine genotoxic and antigenotoxic effects of *C. monspeliensis* leaf extract against mitomycin C (MMC) using an in vitro cytokinesis-block micronucleus assay (CBMN) in the Chinese Hamster Ovarian K1 (CHO-K1) cell line. The phytochemical composition of *C. monspeliensis* extract was evaluated using an untargeted metabolomic approach by employing UPLC-PDA-ESI/MS. The automated in vitro CBMN assay was carried out using image analysis systems with a widefield fluorescence microscope and the ImageStreamX imaging flow cytometer. The phytochemical profile of *C. monspeliensis* extract showed, as the most abundant metabolites, punicalagin, myricetin, gallocathechin, and a labdane-type diterpene. *C. monspeliensis*, at the tested concentrations of 50, 100, and 200 μg/mL, did not induce significant micronuclei frequency, thus indicating the absence of a genotoxic potential. When testing the *C. monspeliensis* extract for antigenotoxicity in the presence of MMC, we observed a hormetic concentration-dependent effect, where low concentrations resulted in a significant protective effect against MMC-induced micronuclei frequency, and higher concentrations resulted in no effect. In conclusion, our findings demonstrate that *C. monspeliensis* extract is not genotoxic and, at low concentration, exhibits an antigenotoxic effect. In relation to this final point, *C. monspeliensis* may act as a potential chemo-preventive against genotoxic agents.

## 1. Introduction

The *Cistus* L. genus (Cistaceae) is predominantly located in the Mediterranean area and comprises several medicinal plants [1]. In Italy, Greece, Spain, and Turkey, various *Cistus* species are utilized as antispasmodic and anti-inflammatory remedies, as well as for general treatment of various skin conditions [2]. Numerous studies have been undertaken on the *Cistus* species, revealing the presence of specific metabolites with anti-inflammatory, antimicrobial, antioxidant, and neuroprotective properties [3,4].

*Cistus monspeliensis* L. (*C. monspeliensis*), also known as “Montpellier rockrose”, is a perennial shrub that thrives in evergreen garrigue vegetation and belongs to the white-flowered Cistus lineage [5]. It is among the most widely distributed *Cistus* species in the Mediterranean region and is utilized as a traditional remedy for treating wounds [1,6]. In Sardinian folk medicine, a compress created from fresh leaves is externally applied for healing wounds, skin disorders, and pain relief; the infusion is also employed for addressing tick bites [7].

Among the various *Cistus* species, *C. monspeliensis* has been found to exhibit the highest antioxidant activity [8]. Studies have shown that the aqueous extract of *C. monspeliensis* could inhibit lipoperoxidation in rat liver microsomes, along with demonstrating antioxidant and superoxide scavenging activity in vitro [9]. Aqueous extracts of *C. monspeliensis* aerial parts have been reported to be endowed with peripheral analgesic effects [5]. The relaxing effects of *C. monspeliensis* aerial parts on isolated vascular and intestinal smooth muscle in vitro also have been established [9]. In human intestinal epithelial cells, *C. monspeliensis* leaf extract induced the expression of enzymes related to intracellular ATP production and boosted intracellular ATP synthesis, suggesting potential antiaging properties [10]. A recent study revealed the anti-inflammatory and antioxidant properties of MeOH/H_2_O (1:1) extracts from the aerial parts and roots of the *C. monspeliensis* plant. It also indicated that the roots exhibited even more potential anti-inflammatory activity compared to the aerial parts, which have been traditionally utilized for this purpose [11].

Research into the phytochemical composition of *C. monspeliensis* has revealed that it contains abundant naturally occurring metabolites, particularly flavonoids, tannins, and terpenoids [12]. The bioactive phytochemical found in the essential oil of *C. monspeliensis* is primarily 13-epi-manoyl oxide, which is considered an intriguing compound in terms of pharmacology. Conversely, the polar extracts contain a high concentration of polyphenols, flavonoids, tannins, and saponins, all possessing antioxidant properties [13]. *C. monspeliensis* aerial parts and root extracts contain comparable levels of the main metabolites, particularly 1-O-methyl-epiinositol. Whereas catechins, gallic acid, and derivatives of pyrogallol are the main ingredients in root extracts, labdane and methoxylated flavonoids are the most distinctive components in the aerial portions [12].

Regarding the toxicity of *C. monspeliensis,* cytotoxic activity of myricetin, extracted with hexane from the aerial parts of the *C. monspeliensis* plant, was reported against nine human leukemic cell lines [14]. Conversely, a diterpene isolated from a hexane extract of *C. monspeliensis* leaves did not display any cytotoxic or cytostatic activity against the same nine cell lines tested [15].

The plant extracts and their constituents are generally recognized as one of the primary sources of bioactive compounds, which are in high demand in a wide range of industries, including food, cosmetics, and medicine. However, their potential to show genotoxic effects, combined with insufficient research on their beneficial activities, significantly restricts their utilization. There is a general belief that plant medicines are safe based on their long-term application; however, some plants used in traditional medicine may also possess genotoxicity [16,17]. For most extracts of plants used in traditional medicine, it is still unknown if they may have genotoxic or antigenotoxic properties. The genotoxic and antigenotoxic studies on *C. monspeliensis* extract are limited in the literature. Some information on the phytochemical composition of the *C. monspeliensis* plant is available. However, information on the genotoxic or antigenotoxic potential of these constituents is lacking, making it useful to screen *C. monspeliensis* extract for genotoxic or antigenotoxic properties. Thus, in the present study, we aimed to evaluate the cytotoxic, genotoxic, and antigenotoxic effects of a methanolic extract of *C. monspeliensis* leaves in vitro using the Chinese Hamster Ovarian K1 (CHO-K1) rodent mammalian cell line in the absence or in the presence of a well-known genotoxic agent, namely mitomycin C (MMC). Genotoxicity evaluation of *C. monspeliensis* extract was carried out using the cytokinesis-block micronucleus assay (CBMN), which is widely employed for assessing DNA damage and evaluating the genotoxicity of chemicals and potential preventive genotoxic and cytotoxic agents [18]. Micronuclei in binucleated cells have been identified as a strong candidate for automation based on image analysis [19]. Indeed, previous studies have documented the automated evaluation of micronuclei through image analysis [20]. In this study, we conducted an automated in vitro CBMN assay using an advanced image analysis approach, combining results obtained from a widefield fluorescence microscope analysis with the those obtained with an ImageStreamX imaging flow cytometer.

## 2. Results

### 2.1. Chemical Characterization of Secondary Metabolites of C. monspeliensis Leaf Extract

**Figure 1:**
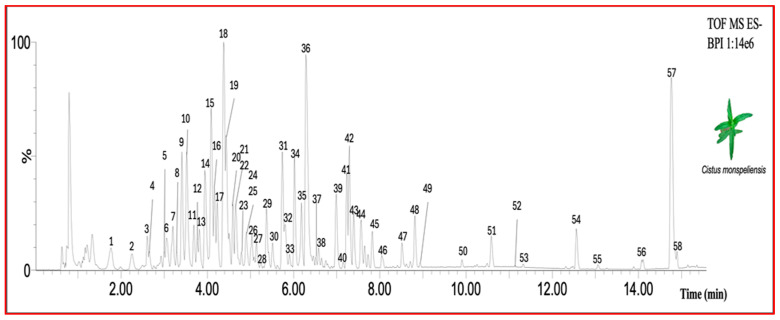
Base peak chromatogram (BPC) of diluted (1:10 *V*/*V*) methanolic leaf extract of *Cistus monspeliensis* in negative ionization mode.

The chemical characterization of secondary metabolites in *C. monspeliensis* leaf extract is shown in Figure 1 and in Table 1. The phytochemical profile of *C. monspeliensis* revealed a large abundance of metabolites (>50) belonging to different chemical classes. In particular, the compounds present in the greatest quantities were found to belong to the class of ellagitannins (punicalagin and punicalagin isomer) and tannins (prodelphinidines and procyanidins), flavonols (in particular, myricetin derivatives), phenolic compounds (mainly galloyl derivatives), and diterpenoids; in particular, a very abundant peak was observed in the final part of the chromatogram (peak 57), probably representing a derivative of labdane diterpenoid, as previously determined in other plant species belonging to the Cistus genus [12]. Moreover, different sulfur-containing compounds were observed in *C. monspeliensis* leaf extracts, although it was not possible to identify them precisely from the ms/ms spectrum of the fragmentation pattern obtained by the FastDDA method. Finally, a benzofuran (Icariside) and two glycolipids (mono- and di-galactosyldiacylglycerol, MGMG and DGMG) were also observed in the full spectra, although they were present in lower amounts compared to the most abundant classes of metabolites.

Table 1 shows in better detail the main characteristics of each numbered peak (retention time, putative annotation, experimental *m*/*z*, molecular formula, main adduct in negative and positive acquisition modes, mass error in negative mode, and the fragments observed in ms/ms).

A diagram showing the percentage of the different phytochemical classes in *C. monspeliensis* methanolic leaf extract is presented in the Supplementary Documents (Supplemental S1 (Figure S1)).

### 2.2. Cytotoxicity and Dose Selection Evolution of C. monspeliensis Extract

**Figure 2:**
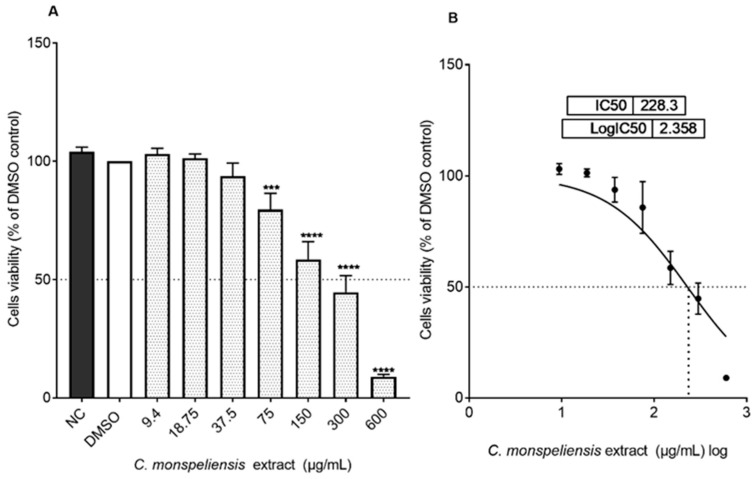
(**A**) Viability (% of DMSO control) of CHO-K1 cells after 24 h incubation with different concentrations of *C. monspeliensis* methanolic extract (9.4–600 µg/mL). Data represent mean ± standard deviation of three independent experiments. (**B**) A nonlinear regression of log-transformed concentration values (curve fit) was applied to determine the IC_50_ value. The percentage of viable cells upon treatment was calculated using this equation: T/C × 100, where T stands for test sample and C for control. Statistical analysis was performed using GraphPad Prism Ver.7. ANOVA followed by Tukey multiple comparison post-test. Different symbols indicate significant differences from DMSO control (*** *p* = 0.0008; **** *p* < 0.0001).

A cytotoxicity evaluation was conducted to determine the appropriate concentrations for the main experiments. Figure 2 (panels A and B) displays the percentages of viable CHO-K1 cells treated with various concentrations of *C. monspeliensis* extract for 24 h, compared to untreated control cells (DMSO control). The data indicate that CHO-K1 cells exposed to *C. monspeliensis* extract for 24 h at concentrations below 75 µg/mL did not demonstrate significant cytotoxicity. However, at concentrations of 75, 150, 300, and 600 µg/mL, a significant decrease in CHO-K1 cell viability was observed, with the decrease being concentration-dependent. At 600 µg/mL, cell viability was less than 10% compared to the untreated control cells. The IC_50_ of the *C. monspeliensis* extract was determined to be 228 µg/mL.

### 2.3. Genotoxicity Assessment Using CBMN Assay

The CBMN assay is widely utilized for assessing DNA damage, evaluating the genotoxicity of chemicals, and potentially preventing genotoxicity and cytotoxicity [19]. The identification of MN in binucleated cells has been identified as a strong candidate for automation based on image analysis [19]. Previous studies have documented the automated evaluation of micronuclei (MN) through image analysis [20,21]. In our research, we conducted the automated in vitro CBMN assay using advanced image analysis systems with a fluorescence microscope and the ImageStreamX Imaging flow cytometer.

#### 2.3.1. Cytotoxicity and Binucleated Cell Evaluation Under the CBMN Assay Analyzed Using Fluorescence Microscope

**Figure 3:**
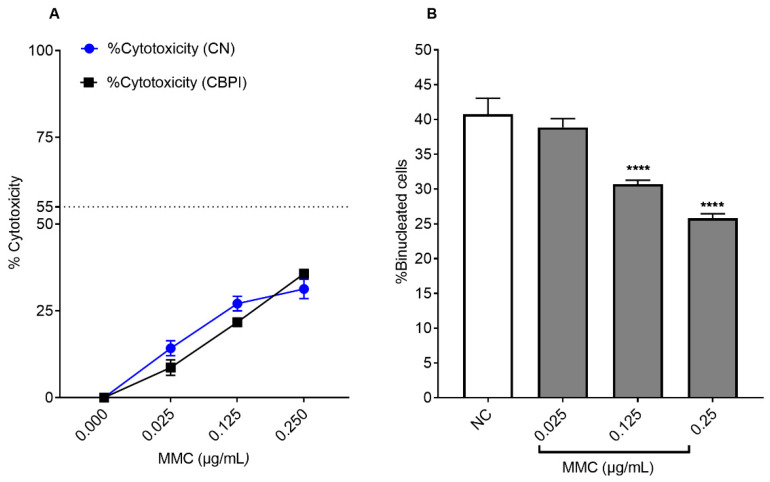
(**A**): Percentage of cytotoxicity (%) CN (blue) and % CBPI (black) in CHO-K1 cells (**B**): % of binucleated cells in CHO-K1 cells after 24 h incubation with different concentrations of MMC, followed by 24 h incubation with 3 μg/mL of cytochalasin B. Graphs represent data collected from three independent experiments. One-way ANOVA, Tukey’s multiple comparisons test using GraphPad Prism 7 software was applied to calculate statistical significance in comparison with NC. (**** *p* < 0.0001).

The well-known genotoxic clastogenic compound MMC induced a dose-dependent increase in cytotoxicity percentage measured by untreated cells (CN), equal to 14.25 ± 2.17, 27.10 ± 2.12, and 31.33 ± 2.78 at 0.025, 0.125, and 0.25 μg/mL, respectively, compared to NC cells (Figure 3A). MMC cytotoxicity also was measured by the cytokinesis-block proliferation index (CBPI) method, based on the principle that cytotoxicity often results in cell-cycle arrest, which is reflected in a decreased ratio of the percentage of binucleate vs. mononucleate cells when using cytochalasin-B (Cyto-B). MMC induced a dose-dependent increase in CBPI cytotoxicity percentage, equal to 8.67 ± 2.25, 21.754 ± 0.71, and 35.6 ± 0.97 at 0.025, 0.125, and 0.25 μg/mL, respectively, compared to NC cells (Figure 3A). Cyto-B was used to block cytokinesis because of its inhibition of actin assembly, thus inducing the formation of binucleated post-mitotic cells. A mean percentage of binucleated cells of 40.75 ± 2.29% was obtained in treating CHO-K1 cells with 3 μg/mL of cytochalasin for 24 h in the NC cells (Figure 3B).

MMC-treated cells at the lowest MMC concentration of 0.025 μg/mL showed no significant differences in binucleated cell formation compared to NC cells. However, a significant reduction in binucleated cell formation was observed in MMC-treated cells at 0.125 and 0.25 μg/mL compared to NC cells. Binucleated cell formation showed 30.7 ± 0.57 and 25.83 ± 0.63 in MMC-treated cells at the 0.125 and 0.25 μg/mL concentrations, respectively, showing good activity of cytochalasin B. The MMC concentration at 0.025 μg/mL was selected for further experiments evaluating the genotoxicity and antigenotoxicity of *C. monspeliensis* extract since the % of binucleated cells was not significant compared to the NC cells, and it showed less cytotoxicity compared to the other MMC concentrations of 0.125 and 0.25 μg/mL.

**Figure 4:**
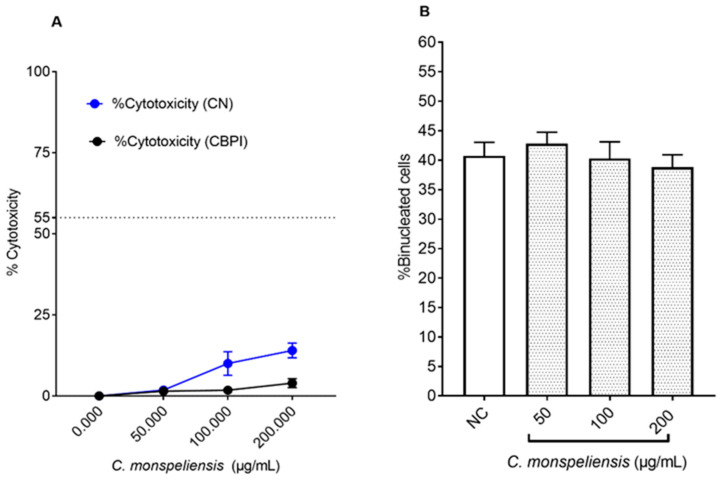
(**A**) Percentage of cytotoxicity (%) CN (blue) and CBPI (black) in CHO-K1 cells; (**B**) % of binucleated cells in CHO-K1 cells after 24 h incubation with different concentrations of *C. monspeliensis* extract, followed by 24 h incubation with 3 μg/mL of cytochalasin B. Graphs represent data collected from three independent experiments. One-way ANOVA, Tukey’s multiple comparisons test using GraphPad Prism 7 software was applied to calculate statistical significance in comparison with NC.

The concentrations of *C. monspeliensis* extract used, namely 50, 100, and 200 μg/mL, induced 1.8 ± 0.92, 10.5 ± 3.62, and 14.36 ± 2.16% of cytotoxicity, respectively (Figure 4A). In addition, the CBPI-based assessment showed less cytotoxicity, of 1.4 ± 0.20, 1.7 ± 0.24, and 3.96 ± 0.73% in cells treated with *C. monspeliensis* extract at 50, 100 and 200 μg/mL, respectively (Figure 4A). No significant differences in the percentage of binucleated cell formation were detected upon treatment with *C. monspeliensis* extract at different concentrations, and the percentage of binucleated cells formed was 42.83 ± 1.92, 40.31 ± 2.81, and 38.83 ± 2.81 in cells with treated with *C. monspeliensis* extract at 50, 100 and 200 μg/mL, respectively, compared to NC (40.75 ± 2.29), indicating that *C. monspeliensis* extract at different concentrations did not affect the cell cycle (Figure 4B).

#### 2.3.2. Micronuclei Scoring Using Fluorescence Microscope and CellProfiler Analysis

**Figure 5:**
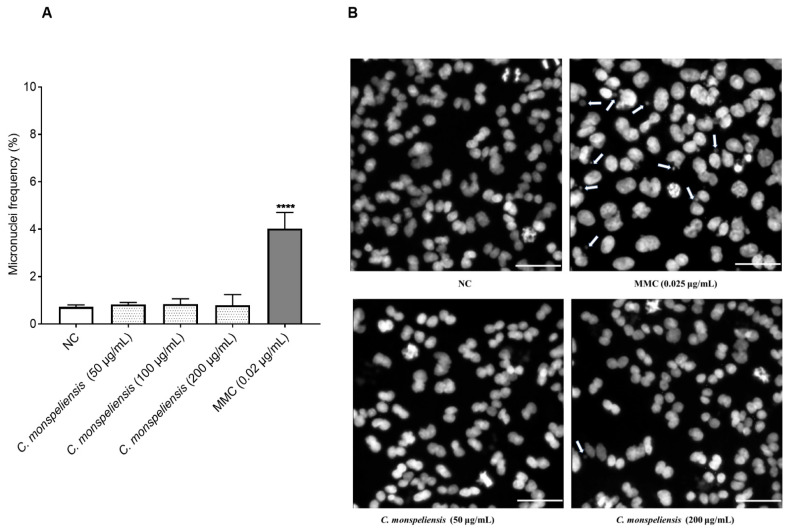
(**A**): Micronuclei frequency in CHO-K1cells after 24 h incubation with three different concentrations of *C. monspeliensis* extract, followed by 24 h incubation with 3 μg/mL of cytochalasin B. Graphs represent data collected from three independent experiments. One-way ANOVA, Tukey’s multiple comparisons test using GraphPad Prism 7 software was applied to calculate statistical significance in comparison with NC (**** *p* < 0.0001). Micronuclei frequency (%) = (binucleated cells with MN/cells × 100). (**B**): Representative microscopic images of micronuclei formation in binucleated CHO-K1 cells with 40× objective after 24 h incubation with NC, MMC and *C. monspeliensis* at 50 and 200 μg/mL. CHO-K1 cell DNA was stained with bisbenzimide (Hoechst dye no. 33258). The white arrows showed the micronuclei. The white line in the image shows the scale bar = 50 µm.

In our study, treating the CHO-K1 cells with the positive control MMC at 0.025 μg/mL for 24 h resulted in a significant increase in micronuclei frequency, to 5.60 ± 0.94% compared to NC control cells (1 ± 0.12%) (Figure 5A). No significant differences in micronuclei frequency were detected upon treatment with *C. monspeliensis* extract at different concentrations when compared to the negative control untreated cells (NC) (Figure 5), thus indicating the absence of a genotoxic potential in the *C. monspeliensis* extract. Micronuclei frequency remained at 1.14 ± 0.02%, 1.17 ± 0.31, and 1.10 ± 0.62% upon 50, 100, and 200 μg/mL *C. monspeliensis* treatment, respectively, compared to NC, which showed 1.00 ± 0.12%. Hence, there was no correlation between induction of cytotoxicity and genotoxicity by *C. monspeliensis* extract in our study. Representative images of micronuclei formation in CHO-K1 cells after 24 h incubation with negative control, MMC at 0.02 μg/mL, and *C. monspeliensis* at 200 μg/mL are shown in Figure 5B.

### 2.4. Anti-Genotoxicity Assessment of C. monspeliensis Extracts Using CBMN

#### 2.4.1. Cytotoxicity Determination Under CBMN

**Figure 6:**
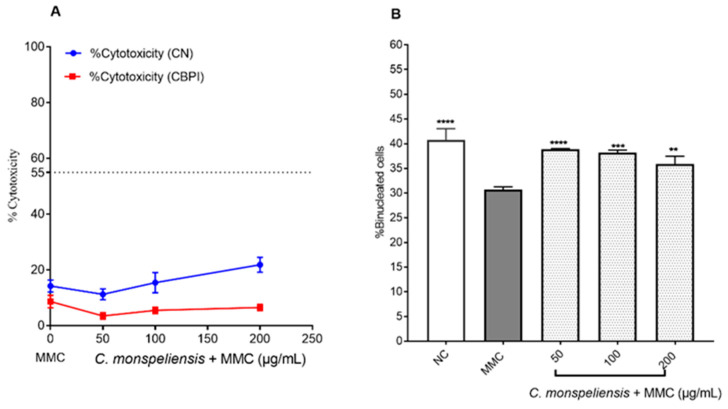
(**A**): Percentage of cytotoxicity (%) CN (blue) and CBPI (red) in CHO-K1 cells. (**B**) % of binucleated cells in CHO-K1 cells after 24 h incubation with different concentrations of *C. monspeliensis* extract in the presence of 0.025 μg/mL MMC followed by 24 h incubation with 3 μg/mL of cytochalasin B. Graphs represent data collected from three independent experiments. One-way ANOVA, Tukey’s multiple comparisons test using GraphPad Prism 7 software was applied to calculate statistical significance in comparison with MMC control. ** *p* = 0.0020, *** *p* = 0.0001, **** *p* < 0.0001.

In another set of experiments, *C. monspeliensis* extract at the same concentrations, namely 50, 100, and 200 μg/mL, was tested for its ability to attenuate MMC-mediated cytotoxic and genotoxic effects on CHOK-1 cells. The cytotoxic activity of *C. monspeliensis* extract at different concentrations with MMC (0.025 μg/mL) was assessed via the determination of CN and CBPI. MMC-treated cells in the presence of *C. monspeliensis* extract at the concentrations of 50 and 100 μg/mL showed no significant differences in the cytotoxicity % determined by the CN method compared to MMC alone. On the other hand, a significant increase in the cytotoxicity % was noticed in MMC-treated cells in combination with *C. monspeliensis* extract at 200 μg/mL (22 ± 2.69%) compared to cells treated with MMC alone (14 ± 2.17%). On the other hand, a significant protective effect of *C. monspeliensis* extract at the lowest concentration of 50 μg/mL against MMC-induced CBPI cytotoxicity was observed (Figure 6A). The % of binucleated cells was found to be significantly higher in the cells treated with *C. monspeliensis* extract in the presence of MMC when compared to cells treated with MMC alone (Figure 6B).

#### 2.4.2. Micronuclei Formation in Binucleated Cells

**Figure 7:**
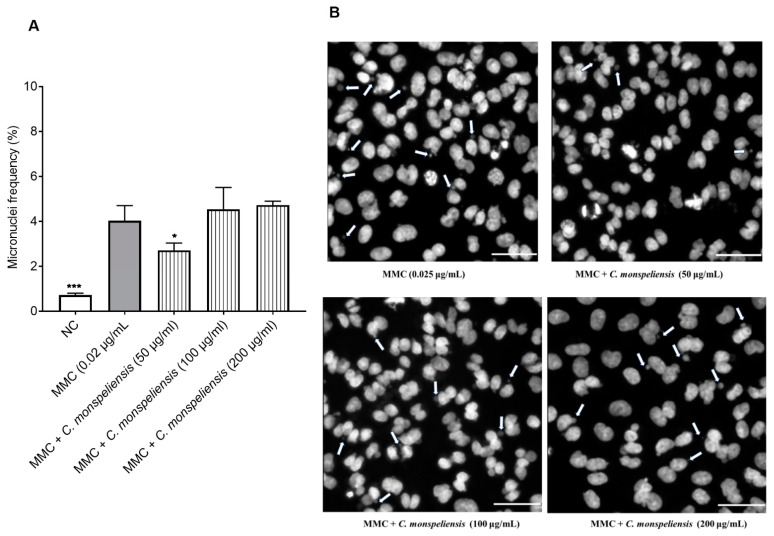
(**A**) Micronuclei frequency in CHO-K1 cells after 24 h incubation with three different concentrations of *C. monspeliensis* extract in the presence of MMC at 0.025 μg/mL, followed by 24 h incubation with 3 μg/mL of cytochalasin B. Graphs represent data collected from three independent experiments. One-way ANOVA, Tukey’s multiple comparisons test using GraphPad Prism 7 software was applied to calculate statistical significance in comparison with MMC control. * *p* < 0.0492, *** *p* = 0.0001. (**B**) Representative microscopic images of for micronuclei formation in binucleated CHO-K1 cells with 40× objective after 24 h incubation with 0.025 μg/mL MMC alone or MMC + *C. monspeliensis* at 5, 100, and 200 μg/mL. CHO-K1 cell DNA was stained with bisbenzimide (Hoechst dye no. 33258). The white arrows showed the micronuclei. The white line in the image shows the scale bar = 50 µm.

Given the absence of genotoxic activity of the *C. monspeliensis* extract, its potential antigenotoxic activity against the genotoxic agent MMC was assessed as well. Micronuclei frequency observed in cells treated with *C. monspeliensis* extract at the concentrations of 100 and 200 μg/mL in combination with MMC was not significantly different (*p* > 0.05) from cells treated with MMC alone (Figure 7A). On the other hand, *C. monspeliensis* extract-treated cells at the lowest concentration of 50 μg/mL in the presence of MMC showed a significantly lower micronuclei frequency by almost 34% compared to MMC control cells, thus indicating a protective effect against MMC-mediated genotoxicity exerted by *C. monspeliensis* extract at 50 μg/mL (Figure 7A). Representative images of micronuclei formation in binucleated CHO-K1 cells treated with *C. monspeliensis* extract in the presence of MMC at 0.025 μg/mL are shown in Figure 7B.

### 2.5. In Vitro CBMN Assay Using the ImagestreamX Imaging Flow Cytometer

**Figure 8:**
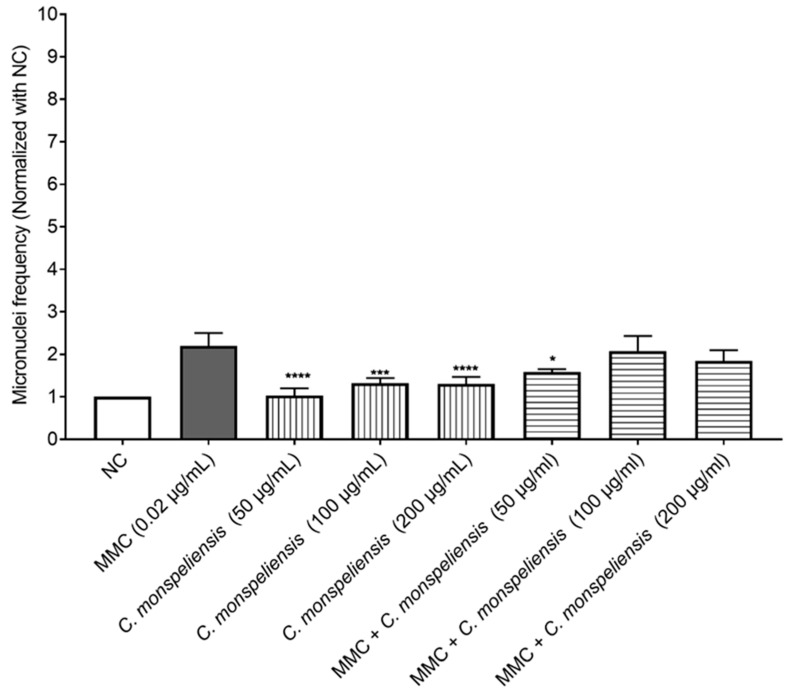
Micronuclei frequency (normalized with NC) per 2000 binucleated CHO-K1cells after 24 h incubation with three different concentrations (50, 100, and 200 μg/mL) of *C. monspeliensis* extract, followed by 24 h incubation with 3 μg/mL of cytochalasin B. Graphs represent data collected from three independent experiments. One-way ANOVA, Tukey’s multiple comparisons test using GraphPad Prism 7 software was applied to calculate statistical significance in comparison with NC control. Micronuclei frequency (%) = (binucleated cells with MN/binucleated cells × 100). * *p* = 0.0192, *** *p* = 0.0001, **** *p* < 0.0001.

**Figure 9:**
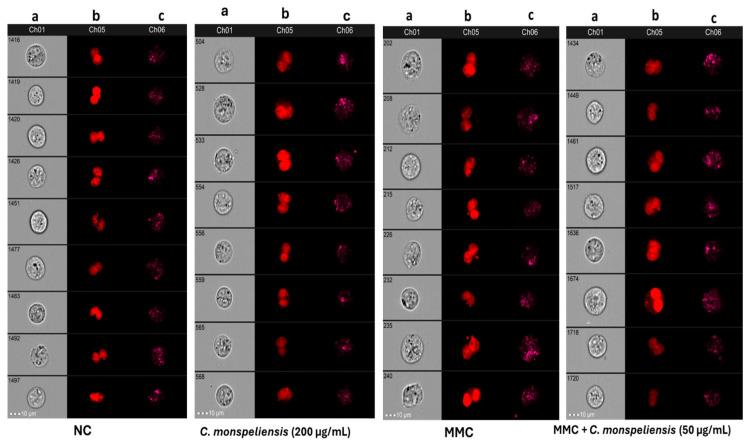
Representative images captured by the ImageStreamX, with 40× objective, that show bright field (**a**) image of single cells, (**b**): Ch05, binucleated cells with micronuclei with or without micronuclei stained with Draq5 of NC, C. monspeliensis extract at tested concentrations of 50 and 200 μg/mL in presence or absence of MMC at 0.025 μg/mL, (**c**) represents side scatter (SSC) image of each cell.

In this set of experiments, micronuclei frequency was analyzed using ImagestreamX with the application of the IDEAS software. As shown in Figure 8, treating CHO-K1 cells with the positive control MMC for 24 h resulted in an increase in micronuclei frequency compared to NC control cells by 2.2 fold. No differences in the percentage of micronuclei frequency were detected upon treatment with *C. monspeliensis* extract at different concentrations compared to NC cells, thus indicating the absence of genotoxic potential in *C. monspeliensis* extract at the tested concentrations. The findings indicate a significant reduction, by 36%, in micronuclei frequency of cells treated with *C. monspeliensis* extract at the concentration of 50 μg/mL compared to cells treated with MMC alone. However, at higher doses of 100 and 200 μg/mL, no significant differences in micronuclei frequency were observed when compared with MMC alone. The findings of MN frequency analyzed by ImagestreamX indicated the same profile of micronuclei frequency obtained following the analysis by fluorescent microscope at the same tested *C. monspeliensis* extract concentrations. Representative images of micronuclei formation in binucleated CHO-K1 cells treated with *C. monspeliensis* extract in the presence of MMC at 0.025 μg/mL are shown in Figure 9.

## 3. Discussion

Medicinal plants have been widely used for treatment in various regions worldwide, with traditional knowledge being passed down through generations based on long-term empirical usage. However, there are limited clinical and experimental data available regarding the safety of most herbal remedies. Assessing the genotoxicity and cytotoxicity of plant extracts is a necessary step in ensuring human safety in terms of potential carcinogenesis and hereditary defects [22]. Specifically, plants of the Cistus genus have been used for a long time as medicines in different countries around the world [23]. Numerous scientific papers, in fact, have shown the presence of various secondary metabolites both in the aerial parts and in the roots of these plant species [11,24], thus suggesting the potential utilization of their extracts as therapeutic agents [25]. The *C. monspeliensis* plant has been widely applied in folk medicine, although its genotoxicity and antigenotoxic effects have not been studied yet. Therefore, this study aimed to investigate the in vitro toxicity, genotoxicity, and antigenotoxicity of *C. monspeliensis* leaf methanolic extract while also characterizing its chemical composition.

This study is part of a larger research initiative conducted by various researchers and collaborators at the Italian National Biodiversity Center, focusing on bioprospecting and bioactivity. The primary aim is to enhance plant-based resources, particularly emphasizing plant-derived secondary bioactive metabolites with novel applications across multiple industrial sectors, including food, pharmaceuticals, cosmetics, and other materials. Our laboratory is engaged in screening of the genotoxic and antigenotoxic properties of medicinal plants found within the Italian flora, with *C. monspeliensis* being one of the plants of our study. As a foundational aspect of this research, we are currently assessing the genotoxicity and antigenotoxicity of *C. monspeliensis*, which is also being examined for other biological activities by different research groups. This investigation will serve as preliminary research to establish the safety profile of this plant.

In this work, the metabolite composition of methanolic extracts obtained from *C. monspeliensis* leaves was evaluated using an untargeted metabolomic approach by UPLC analysis coupled to High Resolution Mass Spectrometry (HRMS). A wide diversity of secondary metabolites was observed; in particular, the most abundant were punicalagin, myricetin, gallocathechyn, and labdane-type diterpen. These metabolites have been reported to have a health benefit. For instance, punicalagin is an ellagitannin, previously described as the most abundant secondary metabolite in pomegranate juice. It has been shown to be able to inhibit cancer cell proliferation and to modulate inflammatory signaling pathways [26,27]. In addition, myricetin possesses a strong antioxidant property and specific pharmacological activities such as hepatoprotective, antitumor, anti-inflammatory, analgesic, and antidiabetic effects [28]. Moreover, gallocatechin, a polyphenolic bioactive component, is endowed with major physiological activities, including anti-inflammatory, antioxidant, and anticancer effects [29,30]. Labdane, belonging to the bicyclic diterpenoid group, has been reported to have a broad spectrum of biological activities, including antimicrobial, antiviral, cytotoxic, radical scavenging, anti-hypertensive, hepatoprotective, and anti-inflammatory properties [15].

In vitro genotoxicity testing has gained significant importance as a tool for the evaluation of the safety of different drugs and chemicals. A compound exhibiting mutagenic activity possesses carcinogenic potential [31]. Micronuclei formation is used as a biomarker for DNA damaging capacity of the tested compound. In this regard, the cytokinesis-blocked micronucleus (CBMN) assay has been frequently used to detect potential genotoxicity and to investigate compounds potentially able to prevent genotoxicity and cytotoxicity [18]. It quantifies the frequency of micronuclei in binucleated cells after adding cytochalasin-B to stop cell division. By using Cyt-B, the scoring of micronuclei is restricted to binucleated cells that have undergone one division and prevents the scoring of unwanted micronuclei resulting from various factors like suboptimal cell culture conditions and changes in cell division rates [32]. Previous studies have documented the automated evaluation of MN through image analysis [20,21]. In our research, we conducted the automated in vitro CBMN assay using advanced image analysis systems with a fluorescent microscope and the ImageStreamX Imaging flow cytometer. The evaluation of cytotoxicity and genotoxicity in this study was performed in CHO-K1 cells, which are commonly used as a reference for genotoxicity and cytotoxicity tests [33], due to their rapid growth rate and stable karyotype of 22 ± 2 chromosomes [34]. These cells are known to be genetically stable, and previous studies have indicated that CHO-KI cells showed 79% sensitivity to positive carcinogenic compounds [35,36]. MMC has been demonstrated to possess genotoxic effects in both in vitro (mammalian cells) and in vivo (animals) studies, and it has been clearly proven to be a carcinogenic substance [37]. Furthermore, the OECD protocol recommends using MMC as a positive control when conducting the CBMN assay [38].

In our preliminary experiments, the screening for a cytotoxic effect of *C. monspeliensis* leaf extract (0–600 μg/mL) on CHO-K1 cells was performed using the MTT assay. Few studies have reported the cytotoxic effects of some *C. monspeliensis* extracts in various cell lines, with different findings based on the cell types used. For instance, the cytotoxic effect of *C. monspeliensis* hexane leaf extract was tested against murine monocyte/macrophages, A-375 human melanoma cells, and MCF7 human breast cancer cells, showing it to be cytotoxic only against A-375 human melanoma cells (IC_50_: 52.44 ± 3.69) [39]. An aqueous extract of *C. monspeliensis* leaves was tested on human intestinal epithelial Caco-2 cells, demonstrating safety and non-cytotoxicity at concentrations ranging from 0.1 to 0.001% (*w*/*v*) [10]. However, there are no available data on the cytotoxic effects of *C. monspeliensis* plant extract on the viability of CHO-K1 cells. Our study found that the crude methanolic extract from *C. monspeliensis* had an IC_50_ of 228 µg/mL after 24 h of incubation.

The examination of various *Cistus* species has revealed differing IC_50_ values, which are contingent upon the specific type of *Cistus* and the cell lines employed. For instance, the methanolic extract of *C. ladanifer* exhibited IC_50_ values of 180.5 ± 0.64, 61.47 ± 0.551, and 144.255 ± 12.43 µg/mL against hepatocellular carcinoma HepG2, human prostate cancer 22Rv1, and human breast cancer MDA-MB-231 cell lines, respectively [40]. Furthermore, the methanolic extract of *C. incanus* demonstrated IC_50_ values of 57.80, 383.61, and 343.40 µg/mL against human malignant melanoma (A375), human squamous cell carcinoma (SCC-15), and non-cancerous human keratinocyte (HaCaT) cell lines, respectively. Additionally, the methanolic extract of *C. ladanifer* showed IC_50_ values of 164.91, >500, and >500 µg/mL in A375, SCC-15, and HaCaT cells, respectively [41]. Therefore, the IC_50_ value obtained from our extract falls within the range reported for other Cistus species.

The OECD guidelines recommend test concentrations ranging from 50 ± 5% cytotoxicity to minimal or no cytotoxicity. Concentrations exceeding 200 µg/mL were found to be toxic against CHOK1 cells, with cytotoxicity levels of more than 50%. Consequently, a concentration of 200 µg/mL was designated as the maximum concentration for this investigation. In addition, concentrations below 50 µg/mL were not toxic against the CHOK1 cells. As a result, cytotoxicity was assessed as part of the MN experiment in the same cells used to score micronuclei, using two separate methods: CN and cytostasis (CBPI%). The CBPI represents the average number of cell cycles per cell upon exposure to cytochalasin B, which should be between 1.5 and 2 cycles. A decrease in CBPI compared to the negative control indicates an inhibition of cell proliferation [42]. As indicated by OECD guidelines, the evaluated chemicals should not have a cytostasis percentage of more than 50% [39]. In our study, the *C. monspeliensis* leaf extract at the highest concentration tested (200 μg/mL) significantly increased cytostasis (23.96 ± 0.73%) compared to the negative control cells, indicating an effect of less than 50% cytotoxicity. As a result, all three selected concentrations, 50, 100 and 200 μg/mL, of *C. monspeliensis* leaf extracts were chosen for MN evaluation. Nevertheless, concentrations below 50 µg/mL were not assessed, which presents an opportunity for future research to explore the effects of concentrations lower than 50 µg/mL.

In our genotoxicity evaluation, the MN frequencies of all *C. monspeliensis* leaf extract-treated groups did not differ significantly from the negative control. Conversely, the positive controls (MMC) displayed significantly increased MN frequencies (*p* < 0.05) compared to NC cells. A clear positive result for the tested chemical can be considered when at least one of the tested concentrations shows a statistically significant increase compared to the concurrent negative control (OECD 20). Additionally, our research has established a pipeline for the automated analysis of MN utilizing CellProfiler for scoring micronuclei with a fluorescence microscope. Moreover, we have introduced the use of innovative features and masks developed within the IDEAS software of the ImageStreamX imaging flow cytometer (IDEAS software version number is 6.2, (Amnis Corporation software, USA)), specifically tailored for enhanced detection of binucleated cells and micronuclei in the ISX-based version of the CBMN assay using CHOK1 cells. With these tools, we created an optimized data analysis template that substantially improved the identification of binucleated cells and increased the frequency of scored micronuclei. To the best of our knowledge, our study is the first one that has implemented this automated analysis approach in CHOK1 cells. The findings from the two analysis methods were not different; indeed, a similar trend of results was obtained in both analyses, where the *C. monspeliensis* extracts in the absence of MMC showed no genotoxic effect and, in the presence of MMC, the lowest concentration produced a significantly lower genotoxic effect when compared to the cells treated with MMC alone.

As stated in the OECD guide, when at least one of the tested concentrations shows a statistically significant increase in comparison to the concurrent negative control under any of the experimental conditions, this can be considered a clear positive result for the tested substance. Hence, our findings indicate that the *C. monspeliensis* methanolic leaf extract did not induce chromosomal damage, since it did not exhibit any genotoxic effect in CHO-K1 cells under the experimental conditions, as confirmed by the in vitro CBMN assay.

Due to the absence of genotoxic activity for the *C. monspeliensis* extract, we also evaluated its potential antigenotoxic activity against the genotoxic agent MMC. Notably, as far as we know, the present study is the first evaluation of the genotoxic or antigenotoxicity potential of *C. monspeliensis* methanolic leaf extract. MMC has been demonstrated as genotoxic in all in vitro and in vivo test systems in mammalian cells and animals, and it has been clearly established as a carcinogenic agent [26]. When testing the *C. monspeliensis* extract for antigenotoxicity, we observed an hormetic concentration-dependent effect, where the lowest concentration resulted in a reduction in micronuclei frequency and the higher concentrations used resulted in no effect. Hormesis, as previously stated, is defined as a stressor agent having a beneficial effect at low dose and an unfavorable effect at higher dose [43]. In general, there are limited studies regarding the genotoxic and antigenotoxic of *Cistus* species, making it difficult to compare our findings with other *Cistus* species findings, except for a few studies. For instance, *Cistus incanus* L. extract inhibited Aflatoxin B1 production by *Aspergillus parasiticus* in macadamia nuts. Aflatoxins are secondary metabolites, with aflatoxin B1 being the most common, reported as carcinogenic, teratogenic and genotoxic [44]. The antigenotoxic hormetic concentration-dependent effect was also observed in other plant extracts, including Dendrobium speciosum stem extract, where lower concentrations of the stem extract showed antigenotoxic effects against 4NQO-induced NDA damage in HEPG2 cells when determined using the comet assay [45]. Additionally, the lowest tested olive mill wastewater extract showed an antigenotoxic hormetic response effect when tested on HepaRG ™ cells using the comet assay [46]. Gentiana lutea extract also showed the hormesis phenomenon, where at lower non-genotoxic doses provided significant protection against 2-amino-3-methyl-3H-imidazo[4,5-f]quinolone and 2-amino-1-methyl-6-phenylimidazo[4,5-b]pyridine (PhIP)-induced genotoxicity in genetically modified Salmonella typhimurium TA1535 when assayed using the SOS/umuC assay [47].

The antigenotoxic effect observed in *C. monspeliensis* extract at the lowest concentration might be due to the presence of punicalagin compounds, being the most abundant molecules identified in the *C. monspeliensis* leaf extract. Punicalagin has been shown to be effective against bleomycin-induced genotoxicity in CHO-K1 cells due to its antioxidative and reactive oxygen species scavenging properties [48]. Furthermore, [49] demonstrated that punicalagin can dramatically reduce benzo[a]pyrene-induced DNA adducts utilizing rat liver microsomal proteins in vitro, and punicalagin protected Salmonella typhimurium DNA from the mutagenic effects of sodium azide, methyl methanesulfonate, benzo[a]pyrene, and 2-aminoflourine. Furthermore, punicalagin showed antigenotoxic properties against cyclophosphamide-induced micronucleated polychromatic mice [43]. Punicalagin also has a strong ability to trigger the DNA repair process [50], and, like a number of other tannins, has the ability to chelate iron ions, which may prevent the Fenton reaction and lessen DNA oxidative stress [50]. Moreover, punicalagin may also form complexes with numerous mutagens, including *C. monspeliensis*, resulting in antigenotoxic activity, given the tannins’ strong attraction for substances like proteins and alkaloids [49]. Myricetin derivatives, including myricetin-3-o-galactoside and myricetin-3-o-rhamnoside, isolated from the leaves of Myrtus communis were found to have an antimutagenic effect by inducing an inhibitory activity against nifuroxazide, aflatoxin B1, and H2O2-induced mutagenicity as measured by the SOS chromotest and comet assay [51]. Gallocatechin isolated from *Paliurus spina-christi* Mill. fruit extracts showed antigenotoxic activities with a protective effect against oxidative DNA damage using the comet assay when evaluated in Chinese hamster lung fibroblast (V79) cell lines [52]. It is well established that the bioactive compounds found in plant extracts can exhibit either synergistic or antagonistic interactions, with multi-target effects being predominant in most cases [53]. In addition, it was concluded that relative concentrations may influence the complex interactions between phytochemicals, which can result in antagonistic, additive, and/or synergistic effects [54]. In our study the *C. monspeliensis* extract showed no antigenotoxic effect at higher concentrations (100–200 µg/mL); this is might be due to antagonistic interactions between bioactive compounds present in the extracts. Further study is needed to confirm this suggestion. However, more studies need to be conducted to better understand the mechanisms underlying the protective effect of the *C. monspeliensis* plant and its main active compounds on other genotoxic agents.

## 4. Materials and Methods

### 4.1. Chemicals

Methanol, HPLC grade, from Merck KGaA (Darmstadt, Germany). Dimethyl sulfoxide (DMSO; 99.9%), cell culture media, medium supplements, and cell culture consumable product, 73% formaldehyde solution (methanol-free) wer purchased from Euroclone Company (Euroclone S.p.A., Milan, Italy). Cytochalasin B and mitomycin C were purchased from D.B.A. Italia s.r.l. (Milan, Italia), 3-(4,5-dimethylthiazol-2-yl) 2,5-diphenyltetrazolium bromide (MTT) was obtained from BioSigma (USA). Hoechst 33342 dye was acquired from Life Technologies. Other mate-rials are described when mentioned.

### 4.2. Plant Materials

#### 4.2.1. Sample Collections

Leaves of *C. monspeliensis* plants were collected at the Botanical Garden of Padua on 5 October 2022 by taking them from a single individual plant, in vegetative state, since it was the only one present at the time of sampling. Three separate and independent pools, each containing almost the same number of leaves (>10), in healthy condition, were prepared, containing at least 5 g of fresh weight material. Leaves were immediately frozen in dry ice to preserve their integrity and subsequently homogenized using an A11 Basic Analytical mill (IKA-Werke, Staufen, Germany); the homogeneous powders were then stored at −80 °C for subsequent use.

#### 4.2.2. Preparation of *C. monspeliensis* Methanolic Leaf Extract

For LC-HRMS analysis, methanolic extracts from each replicate were prepared following the same protocol as previously described [55]. Approximately 1 g of frozen powder for each replicate was extracted by adding 10 volumes of LC-MS grade pure 100% methanol (LC-MS grade; Honeywell, Seelze, Germany), then vortexed for 30 s and sonicated in ice (4 °C) in an ultrasonic bath for 10 min at 40 kHz (SOLTEC, Milano, Italy). Finally, the samples were centrifugated at 4 °C at maximum speed, and the supernatant stored at −80 °C for long-term maintenance. For genotoxicity assessment, 20 g of frozen leaf powder of *C. monspeliensis* leaves was extracted with methanol at 1:10 (*w*/*v*) in closed glass bottles by stirring with a magnetic stirrer for 48 h at room temperature. The methanolic extract was then filtered through quantitative filter paper (ArtiGloss, particle retention in the range of 12–15 μm) and concentrated by rotary evaporation (BUCHI R-210) at 40 °C for 30–50 min. To ensure that all the methanol had evaporated, the extract was placed under a fume hood for 24 h and fully dried with liquid nitrogen. The final dark green viscous extract was transferred into glass amber bottles and stored at 4 °C for subsequent analyses. The extract yield was calculated using the following equation: Yield % = weight of the obtained extract (g)/weight of dried plant sample used (g) × 100. This procedure yielded 2.5% of final methanolic *C. monspeliensis* extract. The concentrated extract was transferred into small glass tubes and stored at 4 °C for subsequent analyses.

### 4.3. Chemical Characterization of C. monspeliensis Methanolic Extract

Three different dilutions in LC-MS grade water (1:10, 1:50, and 1:100, *v*/*v*) were prepared from methanolic extracts of *C. monspeliensis* leaf powder and then filtered through 0.22 mm Minisart filters (Sartorius-Stedim Biotech, Gottingen, Germany) before the LC-HRMS analysis. An untargeted metabolomic approach was carried out by using UPLC-PDA-ESI/MS (Xevo G3-XS gTOF mass platform). Particularly, reverse chromatography was applied using a C18 column (stationary phase) and alternating water (with 0.1% *v*/*v* of formic acid; solvent A) and acetonitrile in mobile phase (solvent B), where concentration changed in a linear way within six different phases. In more detail, the elution gradient started with 1% B, held at 1% B for 1 min, then increased to 40% B at 10 min, to 70% B at 13.5 min, to 90% B at 15 min, and to 99% at 16.5 min. The method was maintained in 99% B for 3.5 min and subsequently decreased to 1% B at 20.1 min. Finally, the method remained in isocratic (1% B) and ended at 25 min. The sample at lower dilution (1:10, *v*/*v*) was also analyzed in FAST-DDA mode (data-dependent analysis) to assist the subsequent analysis with the fragmentation pattern of molecules. Once the data were obtained in the form of a spectrum, the most abundant metabolites corresponding to the main peaks were putatively identified based on different parameters: the accurate mass (from the *m*/*z* ratio of the ions), the retention time (RT), and the fragmentation pattern (ms/ms) of the main detected signals obtained from the FAST-DDA run method, consulting the scientific literature, public databases, and an in silico proprietary library of plant compounds. Two molecules were analyzed using a standard, as we just used 2 standards from our personal library for the identification of catechin and myricitrin (Myricetin-3-O-rhamnoside). For the other molecules, we analyzed the retention time and pattern of fragmentation and used articles from the literature to have confirmation of our putative identification of the molecule. The other compounds were deduced based on the m/z value, retention time, and fragmentation pattern.

### 4.4. Cytotoxicity Determination and Dose Selection of C. monspeliensis Extract

#### 4.4.1. Sample Preparation

The *C. monspeliensis* plant extract was dissolved in DMSO to produce a stock solution at a concentration of 100 mg/mL. The dissolved extract underwent sonication to enhance its solubility and was stored at 4 °C until use.

#### 4.4.2. Cells and Treatment

For the assessment of cytotoxicity and genotoxicity, CHO-K1 cells were utilized. The CHO-K1 cells were obtained from CLS (Cell Lines Service GmbH—Hamburg, Germany). Notably, these cells are commonly used as a standard for genotoxicity and cytotoxicity assessments due to their rapid growth rate and stable karyotype [33,34]. The cells are recognized for their genetic stability, and previous studies have demonstrated that CHO-KI cells display 79% sensitivity to positive carcinogenic chemicals [35].

The cytotoxicity of the *C. monspeliensis* extract was evaluated by the 3-(4,5-dimethylthiazol-2-yl) 2,5-diphenyltetrazolium bromide (MTT) cell viability assay [56], a method for determining cell viability by measuring the mitochondrial dehydrogenase activity. CHO-K1 cells were seeded at a density of 2000 cells/well in 96-well culture plates and allowed to stabilize at 37 °C in a 5% CO_2_ atmosphere for 24 h. Then, the cells were exposed to *C. monspeliensis* extract concentrations in the range of 1–600 μg/mL (2-fold increase) for 24 h. We could not increase the concentration more than 600 μg/mL due to the participation of the extract in the culture medium. *C. monspeliensis* extract was dissolved in DMSO, and the percentage of DMSO was adjusted to be 0.4% in all the used concentrations. Cells treated with the culture medium or DMSO only at 0.4% were used as negative controls in all the experiments. After 24 h incubation, the MTT solution (5 mg/mL) was added for 4 h at 37 °C in a 5% CO_2_ atmosphere. The plate was centrifuged at 1500 rpm for 10 min. The medium was removed and replaced by 100 μL of DMSO. Absorbance was measured at 570 nm after a further 4 h of incubation, when the formazan crystals produced in the MTT reaction were solubilized in DMSO. The percentage of viable cells upon treatment was calculated using the following equation: T/C × 100, where T stands for test sample and C for control. A nonlinear regression of log-transformed concentration values (curve fit) was applied. Statistical analysis was performed using GraphPad Prism Ver.7.

### 4.5. Genotoxicity Study Using Cytokinesis-Block Micronucleus Assay for Fluorescence Analysis

Genotoxic substances can result in the development of micronuclei in cells. It is relatively easy to evaluate micronuclei in interphase cells, so the in vitro micronuclei assay is preferred for evaluating the genotoxic potential of test substances. The CBMN assay was carried out following the instructions by [57], and in accordance with the Organization for Economic Co-operation and Development (OECD No. 487) guideline for testing chemicals [38]. In brief, the cells were exposed to cytochalasin B, a cytokinesis blocker that results in the production of binucleate cells by preventing the separation of daughter cells during mitosis, following their exposure to the test chemicals.

#### 4.5.1. Cell Culture and Treatments

CHO-K1 cells were cultured in Ham’s nutrient F12 medium supplemented with 1% glutamine, 10% fetal bovine serum (FBS), and 1% penicillin/streptomycin. Both *C. monspeliensis* extracts along with the positive MMC control were dissolved in DMSO, and the percentage of DMSO did not exceed 0.4% as the final concentration in both *C. monspeliensis* extracts and MMC. CHO-K1 cells were seeded at a density of 2000/well in 96-well plates (transparent flat-bottom) in a volume of 100 µL of medium and were incubated in a humidified atmosphere at 37 °C with 5% CO2 prior to the addition of the test samples. After 24 h, the cells were treated with *C. monspeliensis* extract at 50, 100, and 200 µg/mL or with MMC (positive control) at 0.025, 0.125, and 0.25 µg/mL. The concentration range was selected based on MTT findings and considering that the highest concentrations should not have more than 55 ± 5% cytotoxicity, as indicated by the OECD. After 24 h incubation with the different tested samples, the media were changed, and the cells were incubated with 3 µg/mL of cytochalasin B (a cytokinesis blocking agent) for an additional 24 h. After 24 h, the medium was removed, and the cells were fixed with 35 µL of formaldehyde (working solution 4%) for 15 min. Then, the fixed solution was discarded, and the cells were washed with phosphate buffered saline (PBS) for 3–5 min twice. Then, the PBS was discarded, and the cells were stained with 100 µL/well of diluted bisbenzimide dye (Hoechst dye) for 30 min in dark conditions at room temperature. Imaging was performed using a Leica DMi8S Inverted Microscope.

As part of the micronucleus experiment, cytotoxicity was assessed in the same cells used for micronuclei scoring using two methods. The first method, which is the percentage of cytotoxicity, was determined based on estimating the reduction in cell number after treatment using the following Equation (1):

(1) $$\%\;Cytotoxicity=100\cdot \frac{\left[\left(number\;of\;umber\;of\;untreated\;cells\right)-\left(number\;of\;umber\;of\;treated\;cells\right)\right]}{number\;of\;untreated\;cells}$$

The second method involves the use of the cytokinesis-block proliferation index (CBPI), which indicates the average number of cell cycles that occur while being exposed to cytochalasin B, and is connected to the reduced ratio of bi-nucleated to mononucleated cells. The cytotoxicity percentage was determined as outlined in Equations (2) and (3).

(2) $$CBPI=100\cdot \frac{\left[N_{1}+2\times N_{2}+3\times N_{3}\right]}{total\;number\;of\;cells}$$

(3) $$\%\;Cytotoxicity\;\left(CBPI\right)=100-\left[100\times \frac{CBPI\;of\;treated\;cells-1}{CBPI\;of\;untreated\;cells-1}\right]$$

where N_1_ is the number of mononucleated cells, N_2_ is the number of binucleated cells, and N_3_ is the number of multinucleated cells.

#### 4.5.2. Fluorescence Microscope Imaging

The images of fixed and DAPI-stained cells were observed under a Leica DMi8S inverted widefield microscope (Wetzlar, Germany) driven by LAS X software (https://www.leica-microsystems.com/products/microscope-software/p/leica-las-x-ls/downloads/ (accessed on 14 November 2024)) and equipped with a Leica DFC 9000GT OS camera and a X-CITE 200DC illuminator. For each condition, an array of 64 adjacent fields of view were acquired using a 40× dry objective (Leica HC PL Fluotar L 40×/0.60), and the DAPI signal was excited and detected with the proper filter set (exc 340–380 nm, dichroic mirror 400 nm, em LP425 nm). Each *C. monspeliensis* concentration had three different wells, where more than 1000 binucleated cells were scored for each concentration per each run, and the experiment was repeated 3 times. Each image was serially numbered and saved.

#### 4.5.3. Automated Detection of Binucleated Cells and Micronuclei Using the CellProfiler Software

The automation of the micronuclei analysis, using the image processing analysis software, can provide a faster and more reliable analysis of the micronucleus assay. Here CellProfiler, an open access cell image analysis software developed by Broad Institute [58], was used for automatic detection of binucleated cells and micronuclei. CellProfiler (version 4.2.6) a freely available modular image analysis software tool capable of handling hundreds of thousands of images and represents a flexible platform for the sharing, testing, and development of new methods by image analysis experts.

CellProfiler uses the concept of a ‘pipeline’ of individual modules; each module processes images in several ways, and the modules are placed in sequential order to create a pipeline. Images acquired with the widefield microscope were first processed with the Leica Lightning deconvolution tool to improve the signal to noise and consequently facilitate recognition of the nuclei and micronuclei by CellProfiler, and then, the images belonging to the same experimental conditions were analyzed simultaneously by CellProfiler.

**Figure 10:**
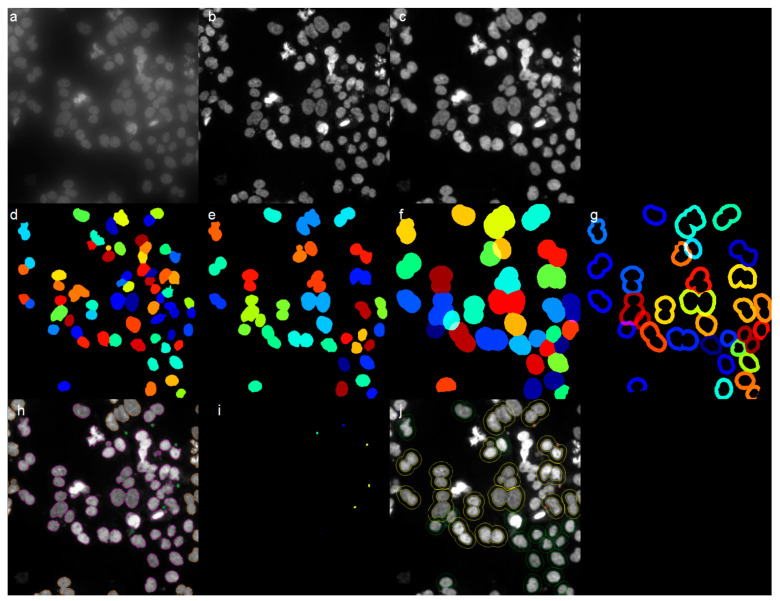
Representative steps for micronuclei analysis using the CellProfiler software (version number 4.2.6): (**a**) Raw image obtained from widefield microscope acquisition. (**b**) Deconvolved image obtained from Leica Lightning software tool. (**c**) Processed image (image crop, smoothing filter, noise-reduction filter) obtained from CellProfiler. (**d**) Nuclei segmentation obtained from CellProfiler. (**e**) Nuclei splitting into classes (mononucleated and binucleated cells) obtained from CellProfiler. (**f**) Definition of cell boundaries (secondary objects), expanding nuclei for a specific distance, obtained from CellProfiler. (**g**) Definition of cell cytoplasm (tertiary objects) obtained from CellProfiler. (**h**) Micronuclei segmentation, obtained from CellProfiler. (**i**) Filtered micronuclei assigned to the nucleus they belong to, obtained from CellProfiler. (**j**) Outlines of mononucleated and binucleated cells overlayed to (**c**).

The pipeline consists of three processing blocks. The first concerns the recognition of nuclei as primary objects, their segmentation, and their splitting into classes (mononucleated, binucleated, and polynucleated cells). Cell clusters due to excessive cell confluency were considered doubtful cases and therefore eliminated. The segmentation was performed based on size (area) and shape (compactness and eccentricity) criteria. The second block concerns the definition of secondary and tertiary objects, i.e., the cells and the cell cytoplasm. Since there is no staining to help locate cell edges, the chosen method is to create an annulus starting from the edges of primary objects, the nuclei, expanding them for a specific distance: the expanded objects are the secondary objects. The cytoplasm is recognized by subtracting the primary objects (nuclei) from that of the secondary objects, so that the remaining doughnut-shaped area represents the cytoplasm. The last block concerns the recognition of micronuclei and their assignment to the nucleus they belong to. Their recognition is based on size (area) and shape (compactness) criteria. Finally, the found micronuclei are assigned to a parent nucleus belonging to the mononucleated or binucleated class. This pipeline allowed us to evaluate the frequency of occurrence of mononucleated and binucleated cells and the frequency of presence of micronuclei for both. The analysis steps are shown in Figure 10 (panels a–j).

### 4.6. Automation of the In Vitro CBMN Using the ImagestreamX Imaging Flow Cytometer

In this part of the experiment, the detection of micronuclei was determined using the ImagestreamX imaging flow cytometer.

#### 4.6.1. Treatment of the Cells

CHO-K1 cells were seeded at a density of 30,000/well in 6-well plates in a volume of 1.5 mL of medium and were incubated in a humidified atmosphere at 37 °C with 5% CO_2_ prior to the addition of the test samples. After 24 h, cells were treated with DMSO at 0.4% (negative control), or with MMC (positive control) at a concentration of 0.02 µg/mL or 0.125 µg/mL, or with *C. monspeliensis* extracts at 50, 100 and 200 µg/mL in the presence or absence of 02 µg/mL MMC. After 24 h incubation with the different treatments, the media were removed and the cells were incubated for an additional 24 h with 3 µg/mL of cytochalasin B. After 24 h, the cells were washed with PBS, detached with Trypsin, and centrifuged at 300× *g* for 3 min. Then, the supernatant was removed, the cell pellets were diluted in 100 µL of PBS without calcium and magnesium in Eppendorf tubes, and all the samples were stained with Draq5 (5 mM, LifeScience Technology, Milan, Italy), which has a high binding affinity for DNA. Draq5 was added to each 100 µL cell suspension to reach a final concentration of 50 mM.

#### 4.6.2. Nucleus and Micronucleus Counting Strategy in IDEAS Software

The ImageStreamX flow cytometer allows a quick capture of single cell images in multiple fluorescence channels. After cell treatment with *C. monspeliensis* extract and MMC, the cells were collected, stained, and then examined. Draq5-stained cells were run in the flow cytometry and acquired by the manufacturer’s software “Inspire”. The acquisition was performed by selecting in focus and single cells, discarding events not representing individual cells. The manufacturer’s software, IDEAS, (version number 6.2, Amnis Corporation software, USA) was then used for the imaging analysis.

**Figure 11:**
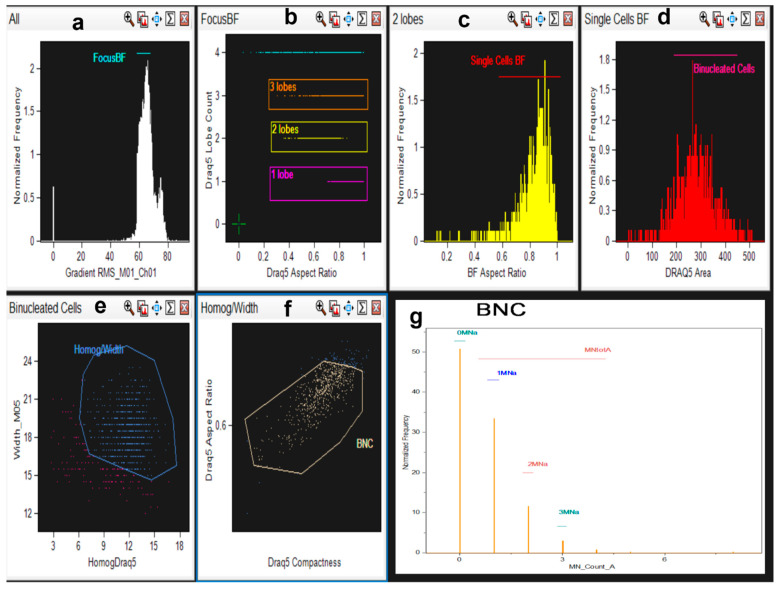
Representative steps of micronuclei analysis using the IDEAS Software (version number 6.2). (**a**) During the analysis, first, selection was based on the Gradient_RMS parameter to confirm in-focus events in the brightfield channel (BF, Ch01). (**b**) A dot plot of Draq5 lobe count versus aspect ratio was created: the reported gates include all cells with two Draq5-stained nuclei, two lobes, single lobe, and cells with more than two nuclei. (**c**) A histogram of BF Aspect Ratio, displaying the gate used to discriminate single cells: all events with Aspect Ratio higher than 0.5 were considered for defining bi-nucleated cells in the histogram reported in (**d**) that used Draq5 Area for this purpose. (**e**) Dot plot of Draq5 Width versus Homogeneity parameters, where the blue gate includes cells with more uniform distribution of Draq5 stain: the majority of the events of interest showed Homogeneity greater than 10 and Width greater than 15. (**f**) Previous Homog/Width population was considered in a dot plot of Draq5 Aspect Ratio intensity versus Draq5-Compactness to finally define the gate that encompasses the acceptable BNC population (yellow gate). Each sample was checked to better define each single gate, evaluating each single event. (**g**) A histogram of our specific spot count feature (micronuclei_Count_A) generated over the masks described in Materials and Methods and in Supplementary Data (2). The linear gates over each bar display the number of BNCs without micronuclei (0 micronuclei) and, respectively, with 1, 2, and 3 micronuclei. The normalized frequency represents the percentage of each type of cell among the total number of cells in the BNC population. BF = brightfield channel, BN = binucleated cells, BNC = binucleated cells with micronuclei.

**Figure 12:**
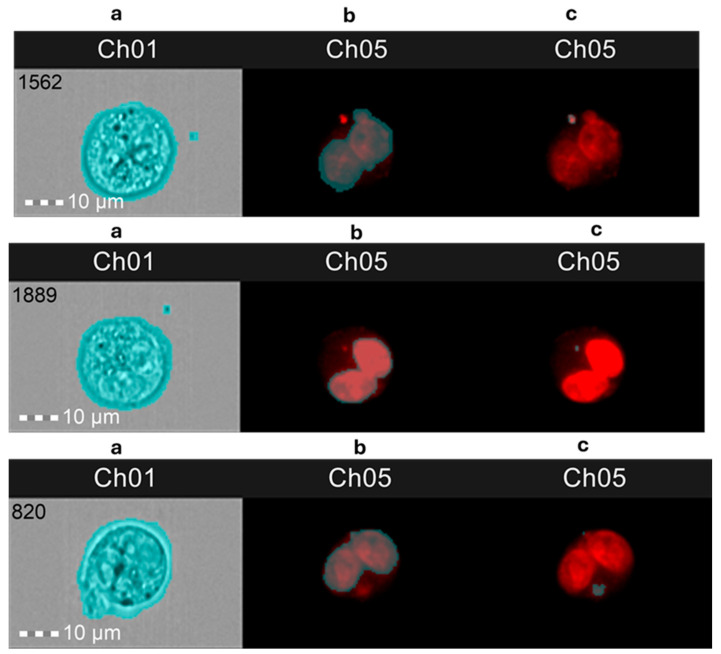
Mask staining (blue shadows over the images): (**a**) Ch01, brightfield (BF) image with the default mask applied that stains all the picture; (**b**) Ch05, binucleated cells with micronuclei (BNC) with a single micronucleus stained with Draq5 with the nuclear mask applied (micronuclei _MaskB_Step3, see Supplementary Data); (**c**) Ch05, BNC with a single micronucleus stained with Draq5 with the complete micronuclei mask applied (micronuclei _MaskA_Step3 and Not micronuclei _MaskB_Step3, see Supplementary Data).

In order to identify and score total cells, binucleated cells, mononucleated cells, multinucleated cells, and micronuclei, an optimized analysis template, defining new features and masks, was developed and created in IDEAS, based on previous literature [59,60,61,62]. Briefly, the following functions were combined through Boolean logic to create the final mask: Threshold, Spot, Range, LevelSet, Dilate. Binucleated cell (BNC) population gates were derived from the strategy reported in Figure 11a–f and, in particular, employing the following parameters: Gradient_RMS (focus), Aspect Ratio, Area, Intensity, Lobe Count, Homogeneity Mean, Compactness. Detailed analysis steps are shown in Figure 11 (panels a–g) and Figure 12. A detail on the development of the new features and image masks for the nucleus and micronucleus counting strategy in IDEAS software (version number 6.2) is presented in Supplementary Materials (Supplemental (S2)).

## 5. Conclusions

The increasing interest in the health advantages linked to various plants and their extracts, along with their economic significance, has led to a heightened demand for comprehensive research, even though there is limited information regarding the genotoxicity of plant extracts that may be detrimental to human health. In the present study, the phytochemical profile as well as the cytotoxic, genotoxic, and antigenotoxic potential of *C. monspeliensis* leaf extract from Italian biodiversity against a well-known genotoxic compound, MMC, using CBMN in the CHO-K1 cell line, were investigated. Our findings reveal that the methanolic extract of *C. monspeliensis* leaf contains a rich variety of metabolites from several chemical classes, such as ellagitannins (including punicalagin and its isomer), tannins (including prodelphinidines and procyanidins), flavonols (especially myricetin derivatives), phenolic compounds (mainly galloyl derivatives), and a labdane-diterpenoid derivative. Regarding the assessment of *C. monspeliensis* genotoxic and antigenotoxic potential, in our work, a pipeline for automated micronuclei analysis using CellProfiler software (version number 4.2.6) with a fluorescence microscope, together with the application of new features and masks developed in the IDEAS software (version number 6.2) of the ImagestreamX imaging flow cytometer for scoring micronuclei in binucleated cells, was developed. Under the chosen experimental conditions, our findings show that *C. monspeliensis* extract at the tested concentrations did not induce a significant increase in micronuclei frequency, thus indicating the absence of a genotoxic potential in CHO-K1 cells. Interestingly, *C. monspeliensis* extract at the lowest tested concentration shows an antigenotoxic effect against a well-known genotoxic agent, MMC, which induced a rise in micronuclei frequency, whereas at the higher used concentrations, no effect was observed, thus indicating a hormetic concentration–dependent effect of *C. monspeliensis* extract. The antigenotoxic effect of *C. monspeliensis* leaf extract could be due to the phytochemicals present in the extract, such as ellagitannins, tannins, flavonols, and phenolic compounds derived from diterpenoids. Nonetheless, additional research is essential to clarify the mechanisms underlying the antigenotoxic effects. Moreover, there is a need for in vivo studies and the identification of the specific phytochemicals involved.

## Figures and Tables

**Table 1:** Chemical molecules putatively identified in methanolic leaf extract of *C. monspeliensis* characterized through HRMS fingerprinting.

	Putative Identification	Retention Time (min)	Elemental Formula	Experimental *m*/*z* (−)	ESI (−) Main Adduct	Mass Error (−), (ppm)	Fragments (−)
1	HHDP-Hex	1.76	C_20_H_18_O_14_	481.0629	[M−H+]−	−2.24503	229.0161; 275.0197; 300.9986
2	Gallic acid-O-Hex	2.25	C_13_H_16_O_10_	331.0667	[M−H+]−	−0.60411	125.0233; 151.0041; 169.0142; 211.0225; 271.0458
3	UI	2.6	-	647.2026	[M−H+]−	-	151.0595
4	Prodelphinidin B2	2.65	C_30_H_26_O_14_	609.123	[M−H+]−	2.298381	125.0233; 177.0204; 305.0674; 423.0743; 441.0827
5	4-hydroxybenzoic acid-4-O-glucoside	3	C_13_H_16_O_8_	345.0844	[M+HCOOH−H+]−	−1.00309	93.0334; 123.0084; 137.0249; 299.0770
6	Prodelphinidin B2 isomer	3.05	C_30_H_26_O_14_	609.123	[M−H+]−	2.298381	-
7	Gallic acid-O-Hex isomer	3.221	C_13_H_16_O_10_	331.0668	[M−H+]−	−0.99055	125.0256; 169.014
8	Prodelphinidin B2 isomer	3.28	C_30_H_26_O_14_	609.123	[M−H+]−	2.298381	-
9	Dihydroxybenzoic acid-O-Hex	3.43	C_13_H_16_O_9_	315.0722	[M−H+]−	−3.67296	108.022; 152.012; 153.018; 315.074
10	Gallocatechin	3.54	C_15_H_14_O_7_	305.0668	[M−H+]−	−2.78206	109.028; 125.023; 137.024; 167.035; 179.033; 219.067; 261.075; 305.066
11	[2-(4,5-Dihydroxy-3-oxocyclohexen-1-yl)-5,7-dihydroxy-4-oxo-2,3-dihydrochromen-3-yl] 3,4,5-trihydroxybenzoate	3.69	C_22_H_18_O_12_	473.0696	[M−H+]−	4.86184	125.0256; 153.0179; 167.035; 319.0483
12	Gallic acid-O-pentoside	3.76	C_12_H_14_O_9_	301.0589	[M+HCOOH−H+]−		169.0142; 168.0072; 149.9972; 125.0256
13	Dihydroxybenzyl alcohol glucoside	3.84	C_13_H_18_O_8_	347.0981	[M−H+]−	−2.32487	124.016; 139.040; 301.093
14	Punicalagin isomer	3.98	C_48_H_28_O_30_	1083.06	[M−2H+]−	−2.23979	300.997; 541.026; 600.992; 781.057; 1083.57
15	Punicalagin-gallate isomer	4.12	C_26_H_26_O_18_	625.0292	[M−H+]−	−1.98593	300.997; 600.987; 603.034; 905.074; 1083.057; 1207.078
16	Dihydroxybenzoic acid-O-arabinoside	4.21	C_12_H_14_O_8_	285.0613	[M−H+]−	−1.18525	108.022; 152.012; 153.024; 285.060
17	bis-HHDP glucose (peduncalagin isomer)	4.28	C_34_H_24_O_22_	783.0698	[M−H+]−	−2.24364	275.019; 481.062
18	Punicalagin gallate isomer	4.42	C_55_H_32_O_35_	625.0291	[M−2H+]−	−1.75626	300.997; 600.987; 603.034; 905.074; 1083.057; 1207.078
19	Punicalagin isomer	4.48	C_48_H_28_O_30_	1083,06	−	−	169.014; 300.997; 541.026; 600.992; 781.057
20	UI (S-containing compound)	4.62	-	323.1165	[M−H+]−	-	96.9592
21	UI (Hex of C6H12O3)	4.66	C_12_H_22_O_8_	293.1249	[M−H+]−	-	131.0725
22	Catechin	4.7	C_15_H_14_O_6_	289.0712	[M−H+]−	0.121204	-
23	UI (Hex of C6H12O3) isomer	4,79	C_12_H_22_O_8_	293,1249	[M−H+]−	-	-
24	Catechin gallate	4.83	C_22_H_18_O_11_	457,0768	[M−H+]−	0.54092	169.014; 305.055
25	UI (S-containing compound)	4.91	-	307.1229	[M−H+]−	-	96.9592
26	UI (putative chalcone derivative)	5.07	C_22_H_26_O_11_	511.1451	[M+HCOOH−H+]−	1.289935	125.0256; 137.0249; 286.0475; 301.0731; 465.1390
27	2-O-acetyl-alpha-D-abequopyranosyl-(1->3)- alpha-D-mannopyranose	5.17	C_14_H_24_O_10_	397.1343	[M+HCOOH−H+]−	-	85.066; 113.062; 157.050; 189.076; 351.1333
28	Vicenin-2	5.24	C_27_H_30_O_15_	593.1501	[M−H+]−	0.842956	297.0769; 353.069; 383.075; 473.1141
29	Epigallocatechin gallate	5.41	C_22_H_18_O_11_	457.0767	[M−H+]−	0.80236	169.014; 305.055
30	UI (S-containing compound isomer)	5.54	-	30.1229	[M−H+]−	-	96.9592
31	Myricetin-O-Hex	5.77	C_21_H_20_O_13_	479.085	[M−H+]−	−5.21831	271.024; 287.019; 316.023; 317.028; 479.085
32	UI (S-containing compound)	5.81	-	30. 1068	[M−H+]−	-	96.9592
33	Ellagic acid arabinoside	5.83	C_19_H_14_O_12_	433.0408	[M−H+]−	−0.55953	299.989; 300.997; 433.042
34	UI (S-containing compound)	6.04	-	457.1172	[M−H+]−	-	245.013; 260.035; 273.042; 287.057; 457.117; 96,9592
35	UI (S-containing compound isomer)	6.2	-	457.1172	[M−H+]−	-	260.035; 457.117; 96,9592
36	Myricetin-3-O-rhamnoside	6,32	C_21_H_20_O_12_	463.088	[M−H+]−	−0.79413	271.024; 287.019; 316.023; 317.028; 463.088
37	Myricetin-O-HExdHex	6.5	C_30_H_26_O_15_	625.1187	[M−H+]−	34.7122	479.0851; 317.0298; 316.0247; 287.0211; 271.029
38	UI (putative lignan)	6.58	C_25_H_32_O_10_	537.1954	[M+HCOOH−H+]−	−4.4789	359.1523; 491.1939
39	Quercetin 3-O-rhamnoside	7.01	C_21_H_20_O_11_	447.0929	[M−H+]−	−0.55733	255.026; 271.024; 300.024; 301.036
40	UI (Putative Rhamnetin derivative)	7.15	-	351.0183	[M−H+]−	-	107.0142; 151.0041; 229.0501; 230.9623; 271.0593
41	Icariside E4	7.27	C_26_H_34_O_10_	551.2138	[M+HCOOH−H+]−	22.88169	299.089; 314.108; 329.139; 341.144; 359.151; 373.227; 505.1958; 551.214
42	UI-Hex of C26H48O8	7.32	C_32_H_58_O_13_	695.3872	[M+HCOOH−H+]−	−3.13408	291.125; 487.327; 649.382
43	UI (S-containing compound)	7,41	-	439,1066	[M−H+]−	-	96.9592; 314.116; 316.023; 317.028; 409.906; 439.107
44	Putative Myricetin-O-Hex-dHex	7.6	C_27_H_30_O_17_	625.1207	[M−H+]−	31.57879	271.024; 316.023; 317.028; 479.085; 625.119
45	UI (S-containing compound)	7.81	-	289.1124	[M−H+]−	-	96.9592
46	Quercetin-O-diHex	8.05	C_30_H_26_O_14_	609.128	[M−H+]−	−5.87729	255.0309; 271.0256; 300.0264; 301.0376; 463.0886
47	Kampferol-O-diHex	8.52	C_30_H_26_O_13_	593.1302	[M−H+]−	−1.18018	227.0349; 255.0309; 284.0364; 285.0410; 447.0953
48	UI	8.85	C_26_H_48_O_8_	533.331	[M+HCOOH−H+]−	−1.23121	487,3276
49	UI	8.93	-	719.3511	[M−H+]−	-	179.0577; 291.2357; 335.2246; 511.2912
50	UI	9.9	C_26_H_30_O_15_	581.1532	[M−H+]−	−4.47388	343.0457; 358.0709; 373.0939
51	5,6,3′-Trihydroxy-7,8,4′-trimethoxyflavone	10.47	C_18_H_16_O_8_	359.0786	[M−H+]−	−5.56984	174.0321; 202.0283; 230.0238; 286.0129; 301.0341; 329.0290
52	Kaempferol-3-O-gal-rham-7-O-rham	11.13	C_39_H_32_O_15_	739.1657	[M−H+]−	0.676438	284.0329; 285.0410
53	Casticin	12.6	C_19_H_18_O_8_	373.0924	[M−H+]−	−0.26282	285.005; 343.048; 358.069
54	DGMG (18:3)	13.06	C_34_H_58_O_16_	721.3616	[M−H+]−	4.158785	235.0833; 277.2168; 397.1346; 415.1508; 675.3598
55	MGMG (18:3)	14.08	C_28_H_48_O_11_	559.3115	[M+HCOOH−H+]−	−9.35114	253.0930; 277.2168; 513,3111
56	UI (putative 3-Hydroxylabda-8(20), 13-dien-15-oic acid)	14.1	C_20_H_32_O_3_	319.2275	[M−H+]−	−0.62651	275,2369
57	UI (putative Diterpenoid of Labdane)	14.8	C_20_H_36_O_3_	323.261	[M−H+]−	−7.4244	263.239; 279.2686
58	UI (Traumatic acid derivative)	14.9	C_45_H_74_O_17_	885.4858	[M−H+]−	-	165.1279; 183.1396; 277.2168; 397.1386

UI = unidentified. Hex = hexoside; dHex = deoxyhexose; Pen = pentoside; S = sulfur.

## Data Availability

Data are contained within the article or Supplementary Materials. The original contributions presented in this study are included in the article/Supplementary Materials. Further inquiries can be directed to the corresponding author.

## Supplementary Materials

The following supporting information can be downloaded at: https://www.mdpi.com/article/10.3390/ijms252413707/s1.
