# Supplementary material for: Phytochemical Profile and In Vitro Cytotoxic, Genotoxic, and Antigenotoxic Evaluation of Cistus monspeliensis L. Leaf Extract
Source: Int J Mol Sci. 2024 Dec 22;25(24):13707. doi: 10.3390/ijms252413707 (PMC11676674; doi:10.3390/ijms252413707)
Supplement: Supplementary file 1 [file ijms-25-13707-s001.zip › ijms-3337499-supplementary.pdf]

# Phytochemical profile, *in Vitro* Cytotoxic, Genotoxic and Antigenotoxic Evaluation of *Cistus Monspeliensis* L., Leaf Extract

**Ghanya Al-Naqeb [1,2,3]*, Gianluca Zorzi [2,4], Amanda Oldani [5], Alberto Azzalin [6], Linda Avesani [2,4], Flavia Guzzo [2,7], Alessia Pascale [8], Rachele De Giuseppe [1,2] and Hellas Cena [1,2,9]**

[1]Laboratory of Dietetics and Clinical Nutrition, Department of Public Health, Experimental and Forensic Medi-cine, University of Pavia, 27100 Pavia; ghanya.al-naqeb@unipv.it; rachele.degiuseppe@unipv.it; hellas.cena@unipv.it
[2]NBFC, National Biodiversity Future Center, 90133 Palermo, Italy;
[3]Department of Food Sciences and Nutrition, Faculty of Agriculture Food and Environment, University of Sana'a, Sana'a, P.O. Box 1247, Yemen
[4]Department of Biotechnology, University of Verona, 37134 Verona, Italy; gianluca.zorzi@univr.it; linda.avesani@univr.it
[5]PASS-Bio Med, Centro Grandi Strumenti, University of Pavia,27100 Pavia, Italy, amanda.oldani@unipv.it
[6]Department of Biology and Biotechnology, University of Pavia, 27100 Pavia, Italy; alberto.azzalin@unipv.it
[7]Department of Biotechnology, University of Verona, 37134 Verona, Italy; flavia.guzzo@univr.it
[8]Department of Drug Sciences, Section of Pharmacology, University of Pavia, Pavia, Italy. alessia.pascale@unipv.it
[9]Clinical Nutrition Unit, ICS Maugeri IRCCS, 27100 Pavia, Italy

## S1. Results Supplemental

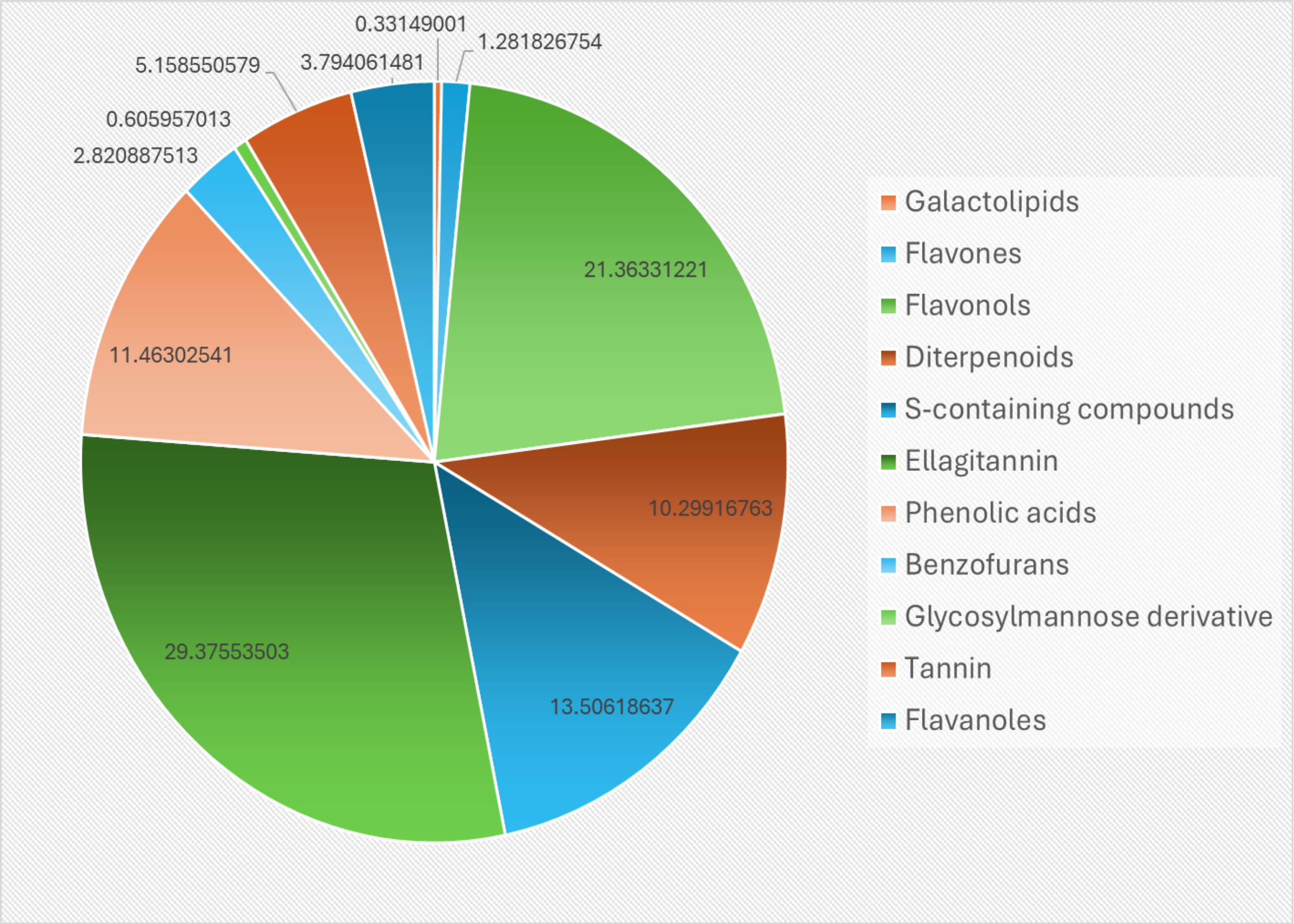


**Figure S1:** A diagram showing the percentage of the different phytochemicals class in *C. monspeliensis* leaves methanolic extract is presented in in Supplementary documents.

**S2. Materials And Methods Supplemental**

*Nucleus and micronucleus counting strategy in IDEAS software*

The ImageStream flow cytometer allows for the quick capture of individual cell images in multiple fluorescence channels. After cell treatment with *C. monspeliensis* extract and MMC, the cells were collected, stained, and then examined. An image capture approach for the acquisition of single cell images for cell samples with concomitant DNA staining using Draq5. The manufacturer's software for the cytometer (IDEAS) was used for the image analysis pipeline.

To filter, identify and score total cells, binucleated cells, mononucleated cells, multinucleated and micronuclei (MN), an optimized analysis template using new features and image masks was developed and created in IDEAS based on previous literature [59-62] with some modification, details analysis step is showing in Fig.2. as following:

"MN_Count_" feature is created using "Spot Count" feature applied to "MN_MASK1" mask. MN_Count_A feature was applied to BNC population deriving from:

"MN_MASK1" mask derives from combining four sub-masks: in particular, "MN_MaskA_Step1" derives from intensity thresholding on the DNA stain channel of 80%; the "MN_MaskA_Step2" derives combining "Spot Mask function" on DNA channel (bright spots between 3-4 pixels) and "MN_MaskA_Step1"; "MN_MaskA_Step3" derives from "Range" function applied to "MN_MaskA_Step2" (2-50 pixels and Aspect Ratio set between 0.4-1.0).

Finally, from "MN_MaskA_Step3" was subtracted the "nuclear mask" created as follows: MN_MaskB_Step3" derives from applying "Range" function to "MN_MaskB_Step2" (set to 250-5000 pixels and Aspect Ratio set between 0.2-1); "MN_MaskB_Step2" derives from applying "Dilate" function to "MN_MaskB_Step1" (dilate set to 1 pixel), while "MN_MaskB_Step1" was produced from "LevelSet" function applied to DNA channel (setting: Middle and 8 pixels).

Boolean logic formula for "MN_MASK1" mask, combined the two masks as follows: MN_MaskA_Step3 and Not MN_MaskB_Step3

where, in details:

MN_MaskA_Step3 = Range (MN_MaskA_Step2, 2-50, 0.4-1)
MN_MaskA_Step2 = MN_MaskA_Step1 and Spot (M05, Ch05, Bright, 4, 3, 1)
MN_MaskA_Step1 = Threshold (M05, Ch05, 80)
MN_MaskB_Step3 = Range (MN_MaskB_Step2, 250-5000, 0.2-1)
MN_MaskB_Step2 = Dilate (MN_MaskB_Step1, 1)
MN_MaskB_Step1 = Level Set (M05, Ch05, Middle, 8)
